# The Prevalence and Diagnostic Ratio of Familial Hypercholesterolemia (FH) and Proportion of Acute Coronary Syndrome in Japanese FH Patients in a Healthcare Record Database Study

**DOI:** 10.1155/2020/5936748

**Published:** 2020-06-11

**Authors:** Tamio Teramoto, Tomohiro Sawa, Satoshi Iimuro, Hyoe Inomata, Takashi Koshimizu, Iori Sakakibara, Katsutoshi Hiramatsu

**Affiliations:** ^1^Teikyo Academic Research Center, Teikyo University, Tokyo, Japan; ^2^Medical Information and Systems Research Center, Teikyo University School of Medicine, Tokyo, Japan; ^3^Innovation and Research Support Center, International University of Health and Welfare, Tokyo, Japan; ^4^Amgen K.K., Tokyo, Japan

## Abstract

**Background:**

Familial hypercholesterolemia (FH) is a genetic disorder characterized by high levels of low-density lipoprotein cholesterol (LDL-C). Because of underdiagnosis, acute coronary syndrome (ACS) is often the first clinical manifestation of FH. In Japan, there are few reports on the prevalence and diagnostic ratios of FH and the proportion of ACS among FH patients in clinical settings.

**Methods:**

This retrospective, observational study used anonymized data from electronic healthcare databases between April 2001 and March 2015 of patients who had ≥2 LDL-C measurements recorded after April 2009. The index date was defined as the date of the first LDL-C measurement after April 2009. The primary endpoint was the prevalence of definite or suspected FH; secondary endpoints included the proportion of FH patients hospitalized for ACS, the proportion of patients using lipid-lowering drugs (LLDs), and LDL-C levels.

**Results:**

Of the 187,781 patients screened, 1547 had definite or suspected FH (0.8%) based on data from the entire period; 832 patients with definite (*n* = 299, 0.16%) or suspected FH (*n* = 533, 0.28%) before the index date were identified in the main analysis cohort. LLDs were used in 214 definite FH patients (71.6%) and 137 suspected FH patients (25.7%). Among definite or suspected FH patients with ACS (*n* = 84) and without ACS (*n* = 748), 32.1% and 30.1% with definite FH and 3.2% and 2.4% with suspected FH had LDL-C levels < 2.6 mmol/L (<100 mg/dL), respectively. Sixty patients (7.2%) were hospitalized due to ACS at the index date.

**Conclusions:**

The prevalence of FH in this Japanese cohort of patients with ≥2 LDL-C measurements at hospitals was 0.8%, which is higher than that currently reported in epidemiological studies (0.2–0.5%). Patients with suspected FH, with or without ACS, had poorly controlled LDL-C levels and were undertreated. The proportion of FH patients who were hospitalized due to ACS was 7.2%.

## 1. Introduction

Familial hypercholesterolemia (FH) is an autosomal dominant genetic disorder with heterozygous and homozygous forms [1]. The underlying cause of FH is a mutation in the gene for the low-density lipoprotein (LDL) receptor or its related genes, such as the genes encoding apolipoprotein B and proprotein convertase subtilisin/kexin-type 9 (PCSK9) [1]. The prevalence of FH varies from study to study. A systematic review and meta-analysis of 19 studies reporting the prevalence of FH found that 1 in 250 people in the general population has this condition [2]. The prevalence of FH in Japan was estimated at between 1 in 200 (0.5%) and 1 in 500 people (0.2%) [3–5].

The principal driver of coronary artery disease (CAD) in FH patients is elevated LDL cholesterol (LDL-C) [6], with a 13-fold increase in the risk of CAD in those not on lipid-lowering drugs (LLDs) [7]. Early diagnosis of FH is necessary for therapy to be effective in reducing the risk of premature CAD [1, 8]. According to the Japan Atherosclerosis Society (JAS) 2017 guidelines, in the absence of secondary hyperlipidemia, fulfilment of ≥2 of the following criteria in patients ≥ 15 years old is diagnostic of FH: hyper-LDL-cholesterolemia (untreated LDL-C of ≥4.7 mmol/L [≥180 mg/dL]); tendon xanthomas on the back of the hands, elbows, or knees, or Achilles tendon hypertrophy or xanthoma tuberosum; or family history of FH or premature CAD (in second-degree relatives of the patient) [4]. The guidelines also mention that a diagnosis of FH should be strongly suspected in patients with LDL-C levels of ≥6.5 mmol/L (≥250 mg/dL) [4], as only 5% of these patients do not have FH [9]. Genetic testing is recommended to confirm the diagnosis of FH in this population [4].

Underdiagnosis of FH is a globally recognized issue [10]. For most undiagnosed FH patients, acute coronary syndrome (ACS) is the first clinical manifestation [11]. It is important to have an early and accurate diagnosis of FH in patients with ACS in order to decide whether aggressive lipid-lowering treatment is necessary. Therefore, we sought to assess the prevalence of FH in Japanese patients and to determine the proportion of FH patients who required hospitalization due to ACS. In addition, we studied the use of LLDs and their effectiveness in this population.

## 2. Methods

### 2.1. Data Source

This was a retrospective observational study using anonymized information on patient demographics, drug prescriptions, comorbidities, and laboratory parameters from cases reported between April 1, 2001, and March 31, 2015 (Supplementary Figure 1). The data were combined from two electronic health record (EHR) databases, the nationwide database developed by the Nomura Research Institute, Ltd. in 2001, which includes data from 70 state and private Japanese hospitals (12 facilities with 1–99 beds, 27 with 100–299 beds, 11 with 300–499 beds, 16 with 500–899 beds, and 4 with ≥900 beds), and the database of the Teikyo University Hospital (Tokyo, Japan). Patients were informed regarding the study and had the right to request that their data should not be used in this study (opt-out style of informed consent).

### 2.2. Patients

Patients with ≥2 LDL-C measurements between April 1, 2009, and March 31, 2015, were identified from the EHR databases. The index date was defined as the date of the first LDL-C measurement during this time period. Based on the index LDL-C measurement, patients were categorized into the following five groups: <1.8 mmol/L (<70 mg/dL), ≥1.8 to <2.6 mmol/L (≥70 to <100 mg/dL), ≥2.6 to <3.6 mmol/L (≥100 to <140 mg/dL), ≥3.6 to <4.7 mmol/L (≥140 to <180 mg/dL), or ≥4.7 mmol/L (≥180 mg/dL) group based primarily on the JAS guidelines [4].

**Figure 1:**
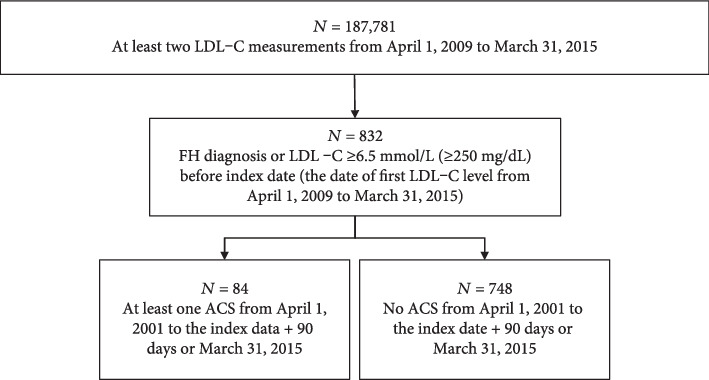
Patient flow. ACS: acute coronary syndrome; FH: familial hypercholesterolemia; LDL-C: low-density lipoprotein cholesterol.

The overall prevalence of FH was based on the number of patients with definite or suspected FH at any time during the entire period between April 1, 2001, and March 31, 2015. Patients with a confirmed diagnosis of FH by a physician, using diagnosis records from the EHR databases, were considered to have definite FH. The remaining patients with ≥1 measured LDL-C level ≥ 6.5 mmol/L (≥250 mg/dL), as per the 2017 JAS guidelines [4], were considered to have suspected FH (FH prevalence cohort). The main study cohort included all patients with definite or suspected FH (per definitions above) prior to the index date (Figure 1).

From this main cohort, patients with comorbid ACS were defined as any patient who had experienced ≥1 ACS between April 1, 2001, and the index date plus 90 days or March 31, 2015, whichever is earlier (Supplementary Figure 1). Among those identified with comorbid ACS, the “hospitalization for ACS cohort” was the subset of patients hospitalized for ACS at the index date.

Comorbidity burden was assessed in the main study cohort between April 1, 2001, and the index date plus 90 days or March 31, 2015, (whichever is earlier) for the six diseases outlined in the Charlson Comorbidity Index (CCI), including CAD, hypertension, diabetes mellitus (DM), noncardiogenic stroke, peripheral artery disease (PAD), and diabetic microangiopathy [12]. The diagnosis of these conditions was based on physician's discretion. LLD use was defined as prescription of an LLD within 90 days prior to the index date.

### 2.3. Endpoints

The primary endpoint of the study was to determine the prevalence of FH (both definite and suspected) in the overall patient population. Secondary endpoints included (1) the diagnostic ratio of definite FH to all FH (definite+suspected), (2) the proportion of patients with FH who were hospitalized for ACS, (3) the proportion of patients with FH using LLDs, (4) the LDL-C levels among patients with FH with and without ACS, and (5) the LDL-C levels of patients according to LLD use.

### 2.4. Statistical Analysis

Demographic and clinical characteristics of patients were summarized using descriptive statistics. Median and interquartile range were calculated for continuous variables, and absolute and relative frequencies were calculated for categorical variables.

The prevalence of FH was calculated as the sum of the number of patients with a definite diagnosis of FH and the number of patients with suspected FH, divided by the total number of patients. The proportion of FH patients with ACS was calculated as the number of patients with hospitalization due to ACS divided by the number of patients with FH, including definite and suspected, before the index date.

Data collection and creation of analysis datasets were performed using R version 3.2.3, and all statistical analyses were performed using SAS 9.4 (SAS Institute Inc., Cary, NC), and *P* values < 0.05 were considered statistically significant.

## 3. Results

### 3.1. FH Prevalence

A total of 187,781 patients had ≥2 LDL-C measurements, and 1547 of these patients had definite or suspected FH between April 1, 2001, and March 31, 2015. The prevalence of FH, including both definite and suspected FH, was 0.8% (*n* = 1547/187,781).

### 3.2. Patient Characteristics and the Proportion of Patients Hospitalized due to ACS

A total of 832 patients with definite (*n* = 299, 0.16% of all patients) or suspected FH (*n* = 533, 0.28% of all patients) prior to the index date were identified (Table 1). The diagnostic ratio of definite FH was 35.9% (*n* = 299/832). Of the 832 patients, 84 had comorbid ACS and 60 patients had been hospitalized for ACS at the index date. Among FH patients, the proportion of patients hospitalized due to ACS was 7.2% (*n* = 60/832).

Women comprised 34.5% of patients with comorbid ACS (*n* = 29) and 55.7% of patients without comorbid ACS (*n* = 417). The median age of patients with or without comorbid ACS was 63.0 years and 60.0 years, respectively, and LLDs were being taken by 71.4% and 38.9% of patients, respectively. CAD, hypertension, and DM were the most common comorbidities both in patients with (95.2%, 76.2%, and 70.2%, respectively) and without (24.1%, 40.4%, and 46.4%, respectively) comorbid ACS.

### 3.3. LDL-C Levels

**Figure 2:**
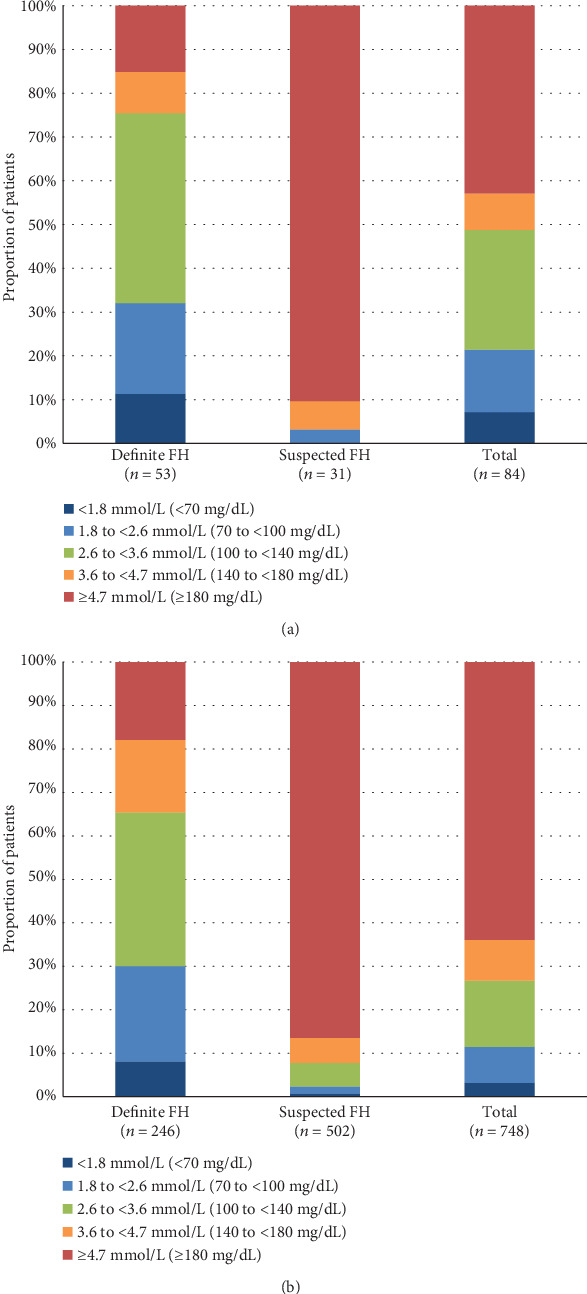
LDL-C levels in patients with definite and suspected FH in (a) patients with ACS and (b) patients without ACS. ACS: acute coronary syndrome; FH: familial hypercholesterolemia; LDL-C: low-density lipoprotein cholesterol.

Among patients with a definite diagnosis of FH and comorbid ACS, 11.3% (*n* = 6/53) had LDL-C levels < 1.8 mmol/L (<70 mg/dL), as defined by the 2017 JAS guidelines [4], whereas among patients with suspected FH and comorbid ACS (*n* = 31), none had LDL-C levels < 1.8 mmol/L (<70 mg/dL) (Figure 2(a)). A similar pattern was observed in patients with definite FH (*n* = 20/246, 8.1%) and suspected FH (*n* = 4/502, 0.8%) who did not have comorbid ACS (Figure 2(b)).

### 3.4. LDL-C Levels according to LLD Use

LLD use was recorded in 71.6% (*n* = 214/299) of patients with definite FH and 25.7% (*n* = 137/533) of patients with suspected FH. Among patients with comorbid ACS, 48.4% (*n* = 15/31) of patients with suspected FH used LLD compared with 84.9% (*n* = 45/53) of patients with definite FH (Table 1).

**Figure 3:**
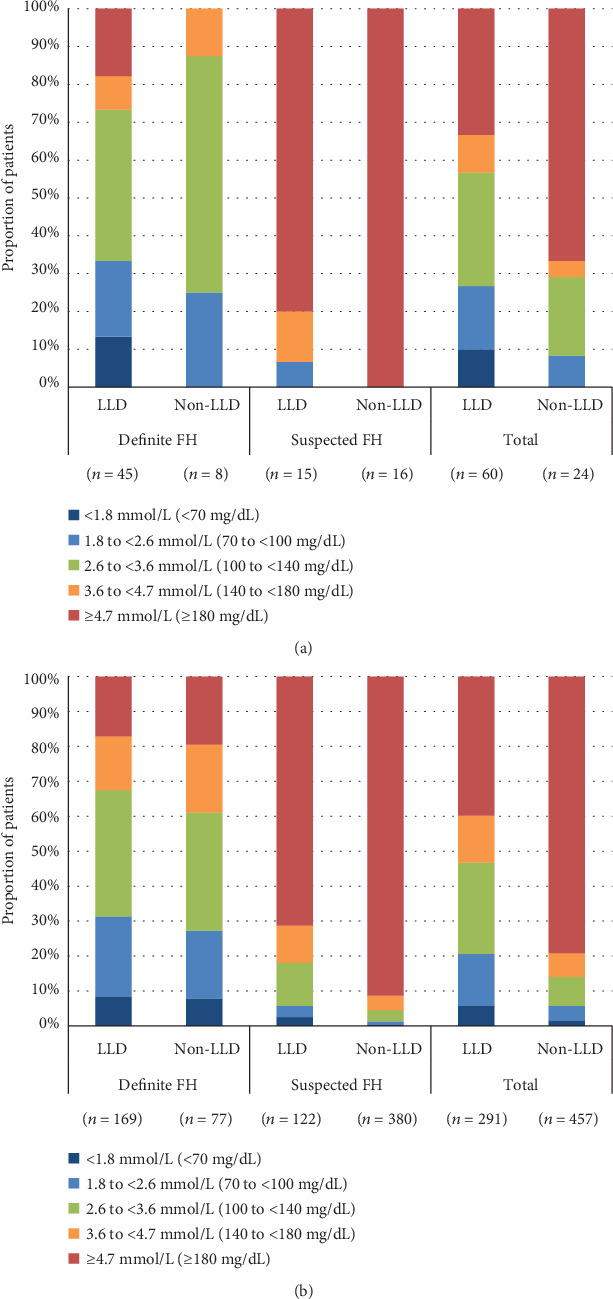
LDL-C levels in patients with definite and suspected FH according to whether or not they were using LLDs in (a) patients with ACS and (b) patients without ACS. FH: familial hypercholesterolemia; LDL-C: low-density lipoprotein cholesterol; LLD: lipid-lowering drug.

Among patients with suspected FH and ACS, those who were taking LLDs had better control of LDL-C levels compared with those who were not (Figure 3(a), Supplementary Table 1). In patients without ACS, control of LDL-C levels was poorer in patients with suspected FH than in those with definite FH (Figure 3(b), Supplementary Table 4). On the other hand, there was little difference in LDL-C levels between patients who were and were not taking LLDs among those with definite FH and no comorbid ACS.

Among overall FH patients with comorbid ACS, those who were taking LLD, regardless of sex or age (<65 or ≥ 65 years), had better control of LDL-C levels compared with those who were not (Figure 3(a), Supplementary Tables 1–3). Among overall FH patients without comorbid ACS, those who were taking LLD regardless of sex or age (<65 or ≥ 65 years) also showed better control of LDL-C levels (Figure 3(b), Supplementary Tables 4–6).

## 4. Discussion

To the best of our knowledge, this was the first study to assess the prevalence of definite or suspected FH, the proportion of FH patients hospitalized due to ACS, and associated management practices in a large sample of in- and outpatients treated in clinical settings in Japan. This study used anonymized data from 187,781 patients, and the data from the FH prevalence cohort were used to estimate the prevalence of FH more accurately. This is considered to have prevented the omission of diagnosed cases due to the lack of data such as LDL-C levels. The results show that the prevalence of FH between 2001 and 2015 was 0.8% (1 in 125 individuals). This value was higher than previous estimates in Japan, such as 1 in 200–500 [3, 5], but similar to the 0.73% (1 in 137) genetically determined prevalence reported in Denmark [7]. A systematic review and meta-analysis of 19 studies conducted in various countries reported that the prevalence of FH in the general population was 0.4% (1 in 250 individuals) [2]. One potential reason for higher prevalence in the present study could be differences between our study population and the general population. Participants of the present study were in- or outpatients with ≥2 LDL-C measurements who were likely to have cardiovascular risk factors or clinical disease severe enough to warrant specialist assessment. However, the results of this study may be more useful for physicians and other healthcare professionals than population-based studies, because they treat similar patients in routine clinical practice. Another potential reason for the higher prevalence of FH in the current study population could be the use of different diagnostic criteria. Previous studies mainly relied on DNA analysis to establish the diagnosis of FH and reported lower estimates for the prevalence of FH compared with studies that used LDL-C measurements (0.40% vs. 0.45%) [2]. Whereas, in the present study, the diagnosis of FH was based on JAS diagnostic criteria for FH [4, 9].

In the present study, 7.2% of patients who had FH at the index date were hospitalized for ACS. Even though this figure is a proportion value, not prevalence, it could be deemed to be a reference value for the prevalence of ACS in patients with FH. The prevalence of ACS in patients with FH also depends on risk factors for ACS, such as increased age, hyperlipidemia, hypertension, diabetes, smoking, obesity, and lack of exercise [13]. To our knowledge, no study has examined the prevalence of ACS in patients with FH, although the prevalence of FH in patients with ACS has been reported in several previous studies. For example, in a study of 4778 Swiss patients with ACS, 1.6% (*n* = 78) had probable/definite FH (Dutch Lipid Clinic Network score > 5) [11]. The prevalence of genetically confirmed FH was 8.7% (*n* = 9) in a study that included 103 patients with ACS who were aged ≤65 years and had LDL-C levels ≥ 4.1 mmol/L (≥160 mg/dL) [14]. In a study of 296 Japanese patients with ACS, the prevalence of FH was 5.7% based on JAS criteria [15]. EXPLORE-J was another study conducted in 1944 Japanese patients with ACS to assess the prevalence of FH using JAS criteria. It reported that 2.7% (*n* = 52) of patients in this population had FH [16]. Although the prevalence of FH in patients with ACS varied widely, it was much higher than that in the general population, highlighting the importance of lipid level control in the prevention of cardiovascular diseases. ACS is a serious condition with a high rate of mortality [17], and its prevalence has been increasing in Japan over recent decades. For example, according to the MIYAGI-AMI registry, the incidence of acute myocardial infarction in the Miyagi prefecture of Japan increased significantly from 7.4 to 27.0 per 100,000 person-years between 1979 and 2008 (*P* < 0.001) [18]. The prevalence of dyslipidemia, as well as hypertension and diabetes, also increased significantly (*P* < 0.01) [18]. Therefore, it is important to identify modifiable risk factors, such as FH, early and treat them effectively to reduce the risk of ACS and its associated sequelae.

Although it is important for patients with FH to achieve target LDL-C values to prevent or delay the development of ACS [19], 37% of the FH patients with comorbid ACS in the present study had suspected FH, but without a confirmed diagnosis despite having abnormally high LDL-C levels. In addition, LDL-C control in patients with suspected FH was poor regardless of the presence of ACS, and patients with definite FH had better LDL-C control with much higher rates of LLD use. This study analyzed LDL-C data between April 1, 2009, and March 31, 2015. At that time, the target LDL-C level was <2.6 mmol/L (<100 mg/dL), as recommended by earlier versions of the JAS guidelines, but the proportion of patients with suspected FH who achieved target LDL-C levels was still poor, even considering the previous target level (definite FH with ACS: 32.1% [*n* = 17/53]; suspected FH with ACS: 3.2% [*n* = 1/31], respectively). Similar results have been reported in patients with suspected FH (LDL-C threshold: ≥4.9 mmol/L [≥190 mg/dL]) in another Japanese study [20]. The mean LDL-C was 3.4 mmol/L (131.3 mg/dL) in patients with suspected FH who were receiving statins and 4.1 mmol/L (157.9 mg/dL) in patients who were receiving non-statin LLDs. LDL-C levels were <3.4 mmol/L (<130 mg/dL) in 39.3% of patients with suspected FH [20]. Although these results cannot be compared directly with our findings because of the different criteria used to categorize LDL-C levels in the two studies, together they highlight a treatment gap in patients with suspected FH.

Among FH patients overall, those who were taking LLDs, regardless of sex or age (<65 or ≥65 years) or the presence or absence of comorbid ACS, had better control of LDL-C levels compared with those who were not taking LLDs. In the clinical setting, administration of LLDs may be more likely to be postponed in premenopausal women, which may affect their LDL-C levels. However, the proportion of women who were taking or not taking LLDs showed little difference in FH patients with comorbid ACS (33.3% and 37.5%, respectively) and in FH patients without comorbid ACS (58.8% and 53.8%, respectively). In addition, since the median age of all patients was around 60.0 years, the proportion of premenopausal women was not high. Therefore, we concluded that there was little influence of sex on LLD prescription bias. LLD administration may also be postponed in elderly patients due to the risk of polypharmacy and declining renal function. The median age of LLD users and non-LLD users was 62.0 and 68.5 years, respectively, among FH patients without comorbid ACS and 60.0 and 59.0 years, respectively, among FH patients with comorbid ACS. Therefore, some influence of age on LLD prescription bias may exist among FH patients without comorbid ACS.

In the present study, the proportion of patients who used LLDs was much lower in patients with suspected FH than in those with definite FH (25.7% vs. 71.6%). The fact that patients on LLDs have been shown to have better control of LDL-C levels, regardless of whether they have a history of ACS, and that patients with suspected FH have been found to have low proportions of LLD usage, emphasizes the importance of a timely FH diagnosis in controlling LDL-C levels. Taken together, these findings highlight the need for more proactive diagnosis and management of FH before patients develop clinical cardiovascular disease, as recommended in current JAS guidelines [4].

The results of this study regarding LDL-C control during treatment are comparable with those observed in previous studies. Treated LDL-C levels were notably higher than target levels (<2.6 mmol/L [<100 mg/dL]) in the SAFEHEART registry (3.0–4.2 mmol/L [116.0–162.1 mg/dL]) [21] and in the CASCADE-FH study (3.5 mmol/L [134 mg/dL]) [22].

Recent guidelines recommend more stringent targets and aggressive therapy for FH patients. The 2017 JAS guidelines introduced a new target LDL-C level of 2.6 mmol/L (100 mg/dL) for primary prevention and 1.8 mmol/L (70 mg/dL) for secondary prevention [4]. The guidelines recommend starting treatment with a statin at the maximum tolerated dose and/or combining a statin with ezetimibe, and if the response is not sufficient, a PCSK9 inhibitor and/or resin and/or probucol should be used [4]. On the other hand, the 2018 Japanese Circulation Society Guidelines on Diagnosis and Treatment of Acute Coronary Syndrome recommends administering strong statins at the maximum tolerated dose [23]. In patients with a diagnosis of FH, lipid-lowering treatment may be started according to the JAS guidelines [4], but in patients without diagnosis, there is a risk of lack or delay of appropriate intervention.

This study had several limitations, including a lack of data from community-based hospitals, inability to ascertain additional relevant data such as family history of FH, history of premature CAD, socioeconomic status, lifestyle-related risk factors, treatment adherence, and potential variations in LDL-C levels based on methodology and measurement errors. Furthermore, as this was a study of in- and outpatients treated in clinical settings at hospital, the prevalence of comorbid disease was expected to be higher than in the general population. The results of this study may be more useful for application to clinical practice than population-based studies. In addition, there was a lack of information on whether genetic analyses were implemented to diagnose FH, the definition of “definite FH” was based only on the physician's diagnosis, and the of “suspected FH” was based on ≥1 LDL-C measurement of ≥6.5 mmol/L (≥250 mg/dL) as per the 2017 JAS guidelines [4]. As a result, it is possible that the number of patients in our FH cohort may have been overestimated and included some patients with similar clinical manifestations, including those with secondary hyperlipidemia. Comparing the results of the present study with those from others, especially non-Japanese, should be approached with caution due to inconsistencies in FH diagnostic criteria between the JAS guidelines [4], the Dutch Lipid Clinic Network criteria [24], the Simon Broome system criteria [25], the Make Early Diagnosis-Prevent Early Death criteria [26], and others.

## 5. Conclusion

The results of the present study show that the prevalence of FH in this Japanese cohort of patients with ≥2 LDL-C measurements at hospitals was 0.8%, which is higher than its currently reported prevalence in epidemiological studies (0.2–0.5%). In addition, the proportion of patients with FH who were hospitalized due to ACS was notably high (7.2%). Patients with suspected FH, with or without ACS, had poorly controlled LDL-C levels and were undertreated. Therefore, improving the diagnostic ratio of FH is important to channel patients to receive appropriate treatments and subsequently have controlled LDL-C levels. Early diagnosis of FH and more aggressive lipid-lowering treatment have the potential to address this issue.

## Figures and Tables

**Table 1:** Demographic and clinical characteristics of definite and suspected FH patients with and without ACS prior to index date.

	Definite FH (*n* = 299)		Suspected FH (*n* = 533)		Total (*n* = 832)	
	With ACS (*n* = 53)	Without ACS (*n* = 246)	With ACS (*n* = 31)	Without ACS (*n* = 502)	With ACS (*n* = 84)	Without ACS (*n* = 748)
Female sex, *n* (%)	16 (30.2)	130 (52.8)	13 (41.9)	287 (57.2)	29 (34.5)	417 (55.7)
Median age, years (IQR)	64.0 (21.0)	61.0 (19.0)	63.0 (14.0)	59.0 (21.0)	63.0 (19.0)	60.0 (20.0)
Taking LLDs, *n* (%)	45 (84.9)	169 (68.7)	15 (48.4)	122 (24.3)	60 (71.4)	291 (38.9)
Previous hospitalization due to ACS, *n* (%)	36 (67.9)	0	24 (77.4)	0	60 (71.4)	0
Comorbidity, *n* (%)						
CAD	50 (94.3)	88 (35.8)	30 (96.8)	92 (18.3)	80 (95.2)	180 (24.1)
Hypertension	45 (84.9)	108 (43.9)	19 (61.3)	194 (38.6)	64 (76.2)	302 (40.4)
DM	40 (75.5)	113 (45.9)	19 (61.3)	234 (46.6)	59 (70.2)	347 (46.4)
Noncardiogenic stroke	12 (22.6)	30 (12.2)	3 (9.7)	54 (10.8)	15 (17.9)	84 (11.2)
PAD	14 (26.4)	23 (9.3)	3 (9.7)	21 (4.2)	17 (20.2)	44 (5.9)
Diabetic microangiopathy	8 (15.1)	44 (17.9)	4 (12.9)	87 (17.3)	12 (14.3)	131 (17.5)

ACS = acute coronary syndrome; CAD = coronary artery disease; DM = diabetes mellitus; IQR = interquartile range; LLD = lipid-lowering drug; PAD = peripheral artery disease.

## Data Availability

The data used to support the findings of this study are available from the corresponding author upon reasonable request.

## Additional Points

*Authorship*. All named authors meet the International Committee of Medical Journal Editors (ICMJE) criteria for authorship for this article, take responsibility for the integrity of the work as a whole, and have given their approval for this version to be published.

## Supplementary Materials

Supplementary Materials Table 1: LDL-C levels in patients with comorbid ACS. ACS = acute coronary syndrome; FH = familial hypercholesterolemia; LDL-C = low-density lipoprotein cholesterol; LLD = lipid-lowering drugs. Table 2: LDL-C Levels in patients with comorbid ACS separated by sex. ACS = acute coronary syndrome; FH = familial hypercholesterolemia; IQR = interquartile range; LDL-C = low-density lipoprotein cholesterol; LLD = lipid-lowering drugs. Table 3: LDL-C levels in patients with comorbid ACS separated by age (<65 or ≥65). ACS = acute coronary syndrome; FH = familial hypercholesterolemia; IQR = interquartile range; LDL-C = low-density lipoprotein cholesterol; LLD = lipid-lowering drugs. Table 4: LDL-C levels in patients without comorbid ACS. ACS = acute coronary syndrome; FH = familial hypercholesterolemia; LDL-C = low-density lipoprotein cholesterol; LLD = lipid-lowering drugs. Table 5: LDL-C levels in patients without comorbid ACS separated by sex. ACS = acute coronary syndrome; FH = familial hypercholesterolemia; IQR = interquartile range; LDL-C = low-density lipoprotein cholesterol; LLD = lipid-lowering drugs. Table 6: LDL-C levels in patients without comorbid ACS separated by age (<65 or ≥65). ACS = acute coronary syndrome; FH = familial hypercholesterolemia; IQR = interquartile range; LDL-C = low-density lipoprotein cholesterol; LLD = lipid-lowering drugs. Figure 1: comorbid ACS and hospitalization for ACS assessment periods. Comorbidities include acute coronary syndrome (ACS), coronary artery disease (CAD), hypertension, diabetes mellitus (DM), noncardiogenic stroke, peripheral artery disease (PAD), and diabetic microangiopathy.

## References

[1] Hovingh G. K., Davidson M. H., Kastelein J. J. P., O'Connor A. M. (2013). Diagnosis and treatment of familial hypercholesterolaemia. *European Heart Journal*.

[2] Akioyamen L. E., Genest J., Shan S. D. (2017). Estimating the prevalence of heterozygous familial hypercholesterolaemia: a systematic review and meta-analysis. *BMJ Open*.

[3] Mabuchi H., Nohara A., Noguchi T. (2011). Molecular genetic epidemiology of homozygous familial hypercholesterolemia in the Hokuriku district of Japan. *Atherosclerosis*.

[4] Kinoshita M., Yokote K., Arai H. (2018). Japan Atherosclerosis Society (JAS) guidelines for prevention of atherosclerotic cardiovascular diseases 2017. *Journal of Atherosclerosis and Thrombosis*.

[5] Goldstein J. L., Hobbs H. H., Brown M. S., Scriver C. R., Beaudet A. L., Sly W. S., Valle D. (2000). Familial hypercholesterolemia. *The Metabolic and Molecular Bases of Inherited Disease*.

[6] Harada-Shiba M., Sugisawa T., Makino H. (2010). Impact of statin treatment on the clinical fate of heterozygous familial hypercholesterolemia. *Journal of Atherosclerosis and Thrombosis*.

[7] Benn M., Watts G. F., Tybjaerg-Hansen A., Nordestgaard B. G. (2012). Familial hypercholesterolemia in the Danish general population: prevalence, coronary artery disease, and cholesterol-lowering medication. *Journal of Clinical Endocrinology and Metabolism*.

[8] Marks D., Thorogood M., Neil H. A. W., Humphries S. E. (2003). A review on the diagnosis, natural history, and treatment of familial hypercholesterolaemia. *Atherosclerosis*.

[9] Teramoto T., Sasaki J., Ishibashi S. (2014). Familial hypercholesterolemia. *Journal of Atherosclerosis and Thrombosis*.

[10] Foody J. M. (2014). Familial hypercholesterolemia: an under-recognized but significant concern in cardiology practice. *Clinical Cardiology*.

[11] Nanchen D., Gencer B., Auer R. (2015). Prevalence and management of familial hypercholesterolaemia in patients with acute coronary syndromes. *European Heart Journal*.

[12] Charlson M. E., Pompei P., Ales K. L., MacKenzie C. R. (1987). A new method of classifying prognostic comorbidity in longitudinal studies: development and validation. *Journal of Chronic Diseases*.

[13] World Health Organization (2009). Global health risks – mortality and burden of disease attributable to selected major risks. https://www.who.int/healthinfo/global_burden_disease/GlobalHealthRisks_report_full.pdf.

[14] Amor-Salamanca A., Castillo S., Gonzalez-Vioque E. (2017). Genetically confirmed familial hypercholesterolemia in patients with acute coronary syndrome. *Journal of the American College of Cardiology*.

[15] Ohmura H., Fukushima Y., Mizuno A. (2017). Estimated prevalence of heterozygous familial hypercholesterolemia in patients with acute coronary syndrome. *International Heart Journal*.

[16] Harada-Shiba M., Ako J., Arai H. (2018). Prevalence of familial hypercholesterolemia in patients with acute coronary syndrome in Japan: results of the EXPLORE-J study. *Atherosclerosis*.

[17] Nakamura M., Yamashita T., Yajima J. (2010). Clinical outcome after acute coronary syndrome in Japanese patients: an observational cohort study. *Journal of Cardiology*.

[18] Takii T., Yasuda S., Takahashi J. (2010). Trends in acute myocardial infarction incidence and mortality over 30 years in Japan: report from the MIYAGI-AMI Registry Study. *Circulation Journal*.

[19] Gencer B., Mach F. (2018). Lipid management in ACS: should we go lower faster?. *Atherosclerosis*.

[20] Teramoto T., Kai T., Ozaki A., Crawford B., Arai H., Yamashita S. (2018). Treatment patterns and lipid profile in patients with familial hypercholesterolemia in Japan. *Journal of Atherosclerosis and Thrombosis*.

[21] de Isla L. P., Alonso R., Mata N. (2016). Coronary heart disease, peripheral arterial disease, and stroke in familial hypercholesterolaemia. *Arteriosclerosis, Thrombosis, and Vascular Biology*.

[22] deGoma E. M., Ahmad Z. S., O’Brien E. C. (2016). Treatment gaps in adults with heterozygous familial hypercholesterolemia in the United States: data from the CASCADE-FH registry. *Circulation Cardiovascular Genetics*.

[23] Kimura K., Kimura T., Ishihara M. (2019). JCS 2018 guideline on diagnosis and treatment of acute coronary syndrome. *Circulation Journal*.

[24] Defesche J., Lansberg P., Umans-Eckenhausen M., Kastelein J. (2004). Advanced method for the identification of patients with inherited hypercholesterolemia. *Seminars in Vascular Medicine*.

[25] Betteridge D. J., Broome K., Durrington P. N. (1991). Risk of fatal coronary heart disease in familial hypercholesterolaemia. Scientific Steering Committee on behalf of the Simon Broome Register Group. *BMJ*.

[26] Williams R. R., Hunt S. C., Schumacher M. C. (1993). Diagnosing heterozygous familial hypercholesterolemia using new practical criteria validated by molecular genetics. *The American Journal of Cardiology*.

