# Supplementary material for: The Prevalence and Diagnostic Ratio of Familial Hypercholesterolemia (FH) and Proportion of Acute Coronary Syndrome in Japanese FH Patients in a Healthcare Record Database Study
Source: Cardiovasc Ther. 2020 Jun 11;2020:5936748. doi: 10.1155/2020/5936748 (PMC7321519; doi:10.1155/2020/5936748)

Supplementary materials

TABLE 1: LDL-C levels in patients with comorbid ACS

LDL-C categories, n (%)	Definite FH		Suspected FH		Overall FH	
	LLD (n=45)	Non-LLD (n=8)	LLD (n=15)	Non-LLD (n=16)	LLD (n=60)	Non-LLD (n=24)
Female sex, n (%)	15 (33.3)	1 (12.5)	5 (33.3)	8 (50.0)	20 (33.3)	9 (37.5)
Median age, years (IQR)	63.0 (20.0)	68.5 (23.0)	59.0 (18.0)	68.5 (16.5)	62.0 (20.0)	68.5 (19.5)
LDL-C categories, n (%)						
<1.8 mmol/L (<70 mg/dL)	6 (13.3)	0	0	0	6 (10.0)	0
1.8 to <2.6 mmol/L (70 to <100 mg/dL)	9 (20.0)	2 (25.0)	1 (6.7%)	0	10 (16.7)	2 (8.3)
2.6 to <3.6 mmol/L (100 to <140 mg/dL)	18 (40.0)	5 (62.5)	0	0	18 (30.0)	5 (20.8)
3.6 to <4.7 mmol/L (140 to <180 mg/dL)	4 (8.9)	1 (12.5)	2 (13.3)	0	6 (10.0)	1 (4.2)
≥4.7 mmol/L (≥180 mg/dL)	8 (17.8)	0	12 (80.0)	16 (100.0)	20 (33.3)	16 (66.7)
No LDL-C data within 90 days before the index date	4 (8.9)	0	2 (13.3)	1 (6.3)	6 (10.0)	1 (4.2)

ACS = acute coronary syndrome; FH = familial hypercholesterolemia; IQR = interquartile range; LDL-C = low-density lipoprotein cholesterol; LLD = lipid-lowering drugs

TABLE 2: LDL-C levels in patients with comorbid ACS separated by sex

LDL-C categories, n (%)	Definite FH				Suspected FH				Overall FH			
	LLD female (n=15)	LLD male (n=30)	Non-LLD female (n=1)	Non-LLD male (n=7)	LLD female (n=5)	LLD male (n=10)	Non-LLD female (n=8)	Non-LLD male (n=8)	LLD female (n=20)	LLD male (n=40)	Non-LLD female (n=9)	Non-LLD male (n=15)
Female sex, n (%)	15 (100)	0	1 (100)	0	5 (100)	0	8 (100)	0	20 (100)	0	9 (100)	0
Median age, years (IQR)	64.0 (21.0)	62.0 (22.0)	77.0 (NA)	65.0 (24.0)	66.0 (10.0)	57.0 (12.0)	68.5 (10.0)	64.0 (23.0)	65.0 (18.0)	59.5 (18.0)	69.0 (12.0)	65.0 (24.0)
LDL-C categories, n (%)												
1.8 to <2.6 mmol/L (70 to <100 mg/dL)	1 (6.7)	8 (26.7)	1 (100.0)	1 (14.3)	0	1 (10.0)	0	0	1 (5.0)	9 (22.5)	1 (11.1)	1 (6.7)
2.6 to <3.6 mmol/L (100 to <140 mg/dL)	9 (60.0)	9 (30.0)	0	5 (71.4)	0	0	0	0	9 (45.0)	9 (22.5)	0	5 (33.3)
3.6 to <4.7 mmol/L (140 to <180 mg/dL)	2 (13.3)	2 (6.7)	0	1 (14.3)	1 (20.0)	1 (10.0)	0	0	3 (15.0)	3 (7.5)	0	1 (6.7)
≥4.7 mmol/L (≥180 mg/dL)	2 (13.3)	6 (20.0)	0	0	4 (80.0)	8 (80.0)	8 (100.0)	8 (100.0)	6 (30.0)	14 (35.0)	8 (88.9)	8 (53.3)
No LDL-C data within 90 days before the index date	2 (13.3)	2 (6.7)	0	0	0	2 (20.0)	1 (12.5)	0	2 (10.0)	4 (10.0)	1 (11.1)	0

ACS = acute coronary syndrome; FH = familial hypercholesterolemia; IQR = interquartile range; LDL-C = low-density lipoprotein cholesterol; LLD = lipid-lowering drugs; NA=not applicable

TABLE 3: LDL-C levels in patients with comorbid ACS separated by age (<65 or ≥65 years)

LDL-C categories, n (%)	Definite FH				Suspected FH				Overall FH			
	LLD age <65y (n=26)	LLD age ≥65y (n=19)	Non-LLD age <65y (n=3)	Non-LLD age ≥65y (n=5)	LLD age <65y (n=11)	LLD age ≥65y (n=4)	Non-LLD age <65y (n=7)	Non-LLD age ≥65y (n=9)	LLD age <65y (n=37)	LLD age ≥65y (n=23)	Non-LLD age <65y (n=10)	Non-LLD age ≥65y (n=14)
Female sex, n (%)	8 (30.8)	7 (36.8)	0	1 (20.0)	2 (11.8)	3 (75.0)	3 (42.9)	5(55.6)	10 (27.0)	10 (43.5)	3 (30.0)	6 (42.9)
Median age, years (IQR)	54.0 (11.0)	75.0 (7.0)	55.0 (5.0)	77.0 (7.0)	57.0 (12.0)	70.5 (9.5)	57.0 (12.0)	73.0 (6.0)	55.0 (12.0)	74.0 (7.0)	55.0 (8.0)	74.0 (9.0)
LDL-C categories, n (%)												
1.8 to <2.6 mmol/L (70 to <100 mg/dL)	3 (11.5)	6 (31.6)	0	2 (40.0)	1 (9.1)	0	0	0	4 (10.8)	6 (26.1)	0	2 (14.3)
2.6 to <3.6 mmol/L (100 to <140 mg/dL)	10 (38.5)	8 (42.1)	3 (100.0)	2 (40.0)	0	0	0	0	10 (27.0)	8 (34.8)	3 (30.0)	2 (14.3)
3.6 to <4.7 mmol/L (140 to <180 mg/dL)	2 (7.7)	2 (10.5)	0	1 (20.0)	2 (18.2)	0		0	4 (10.8)	2 (8.7)	0	1 (7.1)
≥4.7 mmol/L (≥180 mg/dL)	7 (26.9)	1 (5.3)	0	0	8 (72.7)	4 (100.0)	7 (100.0)	9 (100.0)	15 (40.5)	5 (21.7)	7 (70.0)	9 (64.3)
No LDL-C data within 90 days before the index date	2 (7.7)	2 (10.5)	0	0	2 (18.2)	0	0	1 (11.1)	4 (10.8)	2 (8.7)	0	1 (7.1)

ACS = acute coronary syndrome; FH = familial hypercholesterolemia; IQR = interquartile range; LDL-C = low-density lipoprotein cholesterol; LLD = lipid-lowering drugs

TABLE 4: LDL-C levels in patients without comorbid ACS

LDL-C categories, n (%)	Definite FH		Suspected FH		Overall FH	
	LLD (n=169)	Non-LLD (n=77)	LLD (n=122)	Non-LLD (n=380)	LLD (n=291)	Non-LLD (n=457)
Female sex, n (%)	93 (55.0)	37 (48.1)	78 (63.9)	209 (55.0)	171 (58.8)	246 (53.8)
Median age, years (IQR)	61.0 (18.0)	58.0 (17.0)	59.0 (18.0)	59.0 (22.5)	60.0 (18.0)	59.0 (21.0)
LDL-C categories, n (%)						
1.8 to <2.6 mmol/L (70 to <100 mg/dL)	39 (23.1)	15 (19.5)	4 (3.3)	4 (1.1)	43 (14.8)	19 (4.2)
2.6 to <3.6 mmol/L (100 to <140 mg/dL)	61 (36.1)	26 (33.8)	15 (12.3)	12 (3.2)	76 (26.1)	38 (8.3)
3.6 to <4.7 mmol/L (140 to <180 mg/dL)	26 (15.4)	15 (19.5)	13 (10.7)	16 (4.2)	39 (13.4)	31 (6.8)
≥4.7 mmol/L (≥180 mg/dL)	29 (17.2)	15 (19.5)	87 (71.3)	347 (91.3)	116 (39.9)	362 (79.2)
No LDL-C data within 90 days before the index date	45 (26.6)	8 (10.4)	31 (25.4)	31 (8.2)	76 (26.1)	39 (8.5)

ACS = acute coronary syndrome; FH = familial hypercholesterolemia; IQR = interquartile range; LDL-C = low-density lipoprotein cholesterol; LLD = lipid-lowering drugs

TABLE 5: LDL-C levels in patients without comorbid ACS separated by sex

LDL-C categories, n (%)	Definite FH				Suspected FH				Overall FH			
	LLD female (n=93)	LLD male (n=76)	Non-LLD female (n=37)	Non-LLD male (n=40)	LLD female (n=78)	LLD male (n=44)	Non-LLD female (n=209)	Non-LLD male (n=171)	LLD female (n=171)	LLD male (n=120)	Non-LLD female (n=246)	Non-LLD male (n=211)
Female sex, n (%)	93 (100)	0	37 (100)	0	78 (100)	0	209 (100)	0	171 (100)	0	246 (100)	0
Median age, years (IQR)	62.0 (17.0)	59.5 (18.5)	61.0 (15.0)	58.0 (18.0)	61.0 (13.0)	50.0 (21.0)	60.0 (22.0)	57.0 (24.0)	62.0 (16.0)	57.0 (22.0)	60.0 (21.0)	57.0 (21.0)
LDL-C categories, n (%)												
1.8 to <2.6 mmol/L (70 to <100 mg/dL)	16 (17.2)	23 (30.3)	5 (13.5)	10 (25.0)	2 (2.6)	2 (4.5)	2 (1.0)	2 (1.2)	18 (10.5)	25 (20.8)	7 (2.8)	12 (5.7)
2.6 to <3.6 mmol/L (100 to <140 mg/dL)	35 (37.6)	26 (34.2)	12 (32.4)	14 (35.0)	11 (14.1)	4 (9.1)	8 (3.8)	4 (2.3)	46 (26.9)	30 (25.0)	20 (8.1)	18 (8.5)
3.6 to <4.7 mmol/L (140 to <180 mg/dL)	16 (17.2)	10 (13.2)	13 (35.1)	2 (5.0)	9 (11.5)	4 (9.1)	9 (4.3)	7 (4.1)	25 (14.6)	14 (11.7)	22 (8.9)	9 (4.3)
≥4.7 mmol/L (≥180 mg/dL)	20 (21.5)	9 (11.8)	6 (16.2)	9 (22.5)	55 (70.5)	32 (72.7)	190 (90.9)	157 (91.8)	75 (43.9)	41 (34.2)	196 (79.7)	166 (78.7)
No LDL-C data within 90 days before the index date	27 (29.0)	18 (23.7)	5 (13.5)	3 (7.5)	23 (29.5)	8 (18.2)	17 (8.1)	14 (8.2)	50 (29.2)	26 (21.7)	22 (8.9)	17 (8.1)

ACS = acute coronary syndrome; FH = familial hypercholesterolemia; IQR = interquartile range; LDL-C = low-density lipoprotein cholesterol; LLD = lipid-lowering drugs

TABLE 6: LDL-C levels in patients without comorbid ACS separated by age (<65 or ≥65 years)

LDL-C categories, n (%)	Definite FH				Suspected FH				Overall FH			
	LLD age<65 (n=100)	LLD age≥65 (n=69)	Non-LLD age<65 (n=53)	Non-LLD age≥65 (n=24)	LLD age<65 (n=80)	LLD age≥65 (n=42)	Non-LLD age<65 (n=239)	Non-LLD age≥65 (n=141)	LLD age<65 (n=180)	LLD age≥65 (n=111)	Non-LLD age<65 (n=292)	Non-LLD age≥65 (n=165)
Female sex, n (%)	51 (51.0)	42 (60.9)	25 (47.2)	12 (50.0)	47 (58.8)	31 (73.8)	121 (50.6)	88 (62.4)	98 (54.4)	73 (65.8)	146 (50.0)	100 (60.6)
Median age, years (IQR)	53.0 (14.5)	71.0 (7.0)	55.0 (15.0)	70.5 (8.0)	54.0 (14.5)	72.0 (7.0)	50.0 (18.0)	72.0 (9.0)	53.0 (14.0)	71.0 (7.0)	51.0 (17.5)	72.0 (8.0)
LDL-C categories, n (%)												
1.8 to <2.6 mmol/L (70 to <100 mg/dL)	17 (17.0)	22 (31.9)	11 (20.8)	4 (16.7)	1 (1.3)	3 (7.1)	1 (0.4)	3 (2.1)	18 (10.0)	25 (22.5)	12 (4.1)	7 (4.2)
2.6 to <3.6 mmol/L (100 to <140 mg/dL)	39 (39.0)	22 (31.9)	16 (30.2)	10 (41.7)	10 (12.5)	5 (11.9)	6 (2.5)	6 (4.3)	49 (27.2)	27 (24.3)	22 (7.5)	16 (9.7)
3.6 to <4.7 mmol/L (140 to <180 mg/dL)	12 (12.0)	14 (20.3)	9 (17.0)	6 (25.0)	8 (10.0)	5 (11.9)	7 (2.9%)	9 (6.4)	20 (11.1)	19 (17.1)	16 (5.5)	15 (9.1)
≥4.7 mmol/L (≥180 mg/dL)	23 (23.0)	6 (8.7)	14 (26.4)	1 (4.2)	59 (73.8)	28 (66.7)	224 (93.7)	123 (87.2)	82 (45.6)	34 (30.6)	238 (81.5)	124 (75.2)
No LDL-C data within 90 days before the index date	25 (25.0)	20 (29.0)	5 (9.4)	3 (12.5)	17 (21.3)	14 (33.3)	21 (8.8)	10 (7.1)	42 (23.3)	34 (30.6)	26 (8.9)	13 (7.9)

ACS = acute coronary syndrome; FH = familial hypercholesterolemia; IQR = interquartile range; LDL-C = low-density lipoprotein cholesterol; LLD = lipid-lowering drugs

FIGURE 1: *Comorbid ACS and Hospitalization for ACS Assessment Periods*

Comorbidities include acute coronary syndrome (ACS), coronary artery disease (CAD), hypertension, diabetes mellitus (DM), non-cardiogenic stroke, peripheral artery disease (PAD) and diabetic microangiopathy.

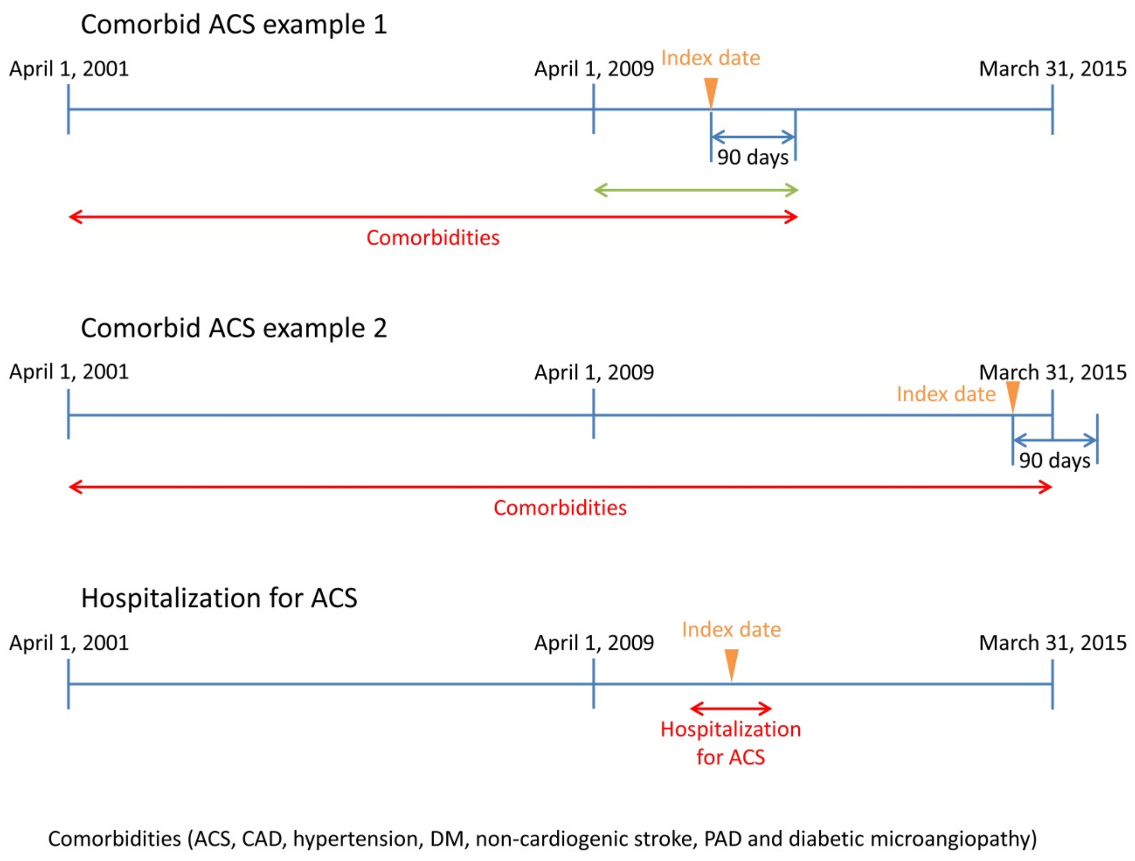

Supplement: Supplementary Materials — Table 1: LDL-C levels in patients with comorbid ACS. ACS = acute coronary syndrome; FH = familial hypercholesterolemia; LDL-C = low-density lipoprotein cholesterol; LLD = lipid-lowering drugs. Table 2: LDL-C Levels in patients with comorbid ACS separated by sex. ACS = acute coronary syndrome; FH = familial hypercholesterolemia; IQR = interquartile range; LDL-C = low-density lipoprotein cholesterol; LLD = lipid-lowering drugs. Table 3: LDL-C levels in patients with comorbid ACS separated by age (<65 or ≥65). ACS = acute coronary syndrome; FH = familial hypercholesterolemia; IQR = interquartile range; LDL-C = low-density lipoprotein cholesterol; LLD = lipid-lowering drugs. Table 4: LDL-C levels in patients without comorbid ACS. ACS = acute coronary syndrome; FH = familial hypercholesterolemia; LDL-C = low-density lipoprotein cholesterol; LLD = lipid-lowering drugs. Table 5: LDL-C levels in patients without comorbid ACS separated by sex. ACS = acute coronary syndrome; FH = familial hypercholesterolemia; IQR = interquartile range; LDL-C = low-density lipoprotein cholesterol; LLD = lipid-lowering drugs. Table 6: LDL-C levels in patients without comorbid ACS separated by age (<65 or ≥65). ACS = acute coronary syndrome; FH = familial hypercholesterolemia; IQR = interquartile range; LDL-C = low-density lipoprotein cholesterol; LLD = lipid-lowering drugs. Figure 1: comorbid ACS and hospitalization for ACS assessment periods. Comorbidities include acute coronary syndrome (ACS), coronary artery disease (CAD), hypertension, diabetes mellitus (DM), noncardiogenic stroke, peripheral artery disease (PAD), and diabetic microangiopathy. [file 5936748.f1.pdf]
